# Dedifferentiated liposarcoma of the spermatic cord: Case report and review of literature

**DOI:** 10.1016/j.ijscr.2020.06.051

**Published:** 2020-06-12

**Authors:** Youssef Shaban, Adel Elkbuli, David Kim, Alia Abdulla, Dessy Boneva, Mark McKenney, Jason Wolf

**Affiliations:** a Department of Surgery, Kendall Regional Medical Center, Miami, FL, USA; b University of South Florida, Tampa, FL, USA; c Department of Pathology, University of Texas Health Science at San Antonio (UT Health San Antonio), San Antonio, TX, USA; d Department of Surgical Oncology, Aventura Hospital and Medical Center, Aventura, FL, USA; e Department of Urology, Kendall Regional Medical Center, Miami, FL, USA

**Keywords:** Liposarcoma, Radical orchiectomy, Surgical oncology, Patients centered outcomes

## Abstract

**Introduction:**

Malignant spermatic cord tumors have an annual incidence of 0.3 cases/million. The vast majority of tumors in this region are benign. We present a rare case of a dedifferentiated liposarcoma of the spermatic cord successfully treated.

**Presentation of case:**

A 59-year-old gentleman presented complaining of an enlarging painful right groin mass. On exam there was an obvious 10 cm inguinal mass. Imaging illustrated a right inguinal soft tissue mass that was not present on imaging 22 months prior.

The patient underwent a right inguinal exploration, en bloc resection of the mass, and radical orchiectomy to ensure negative margins. Histopathological analysis revealed a grade 2 dedifferentiated liposarcoma that measured 9 × 6 × 5 cm, with 5 cm negative margins. The patient did well and was discharged on postoperative day one. On 6-month follow-up there was no evidence of recurrence.

**Discussion:**

We present a rare dedifferentiated liposarcoma of the spermatic cord that was successfully treated with surgical resection. This case highlights the importance of maintaining a high index of suspicion coupled with a thorough history and physical examination when encountering an enlarging inguinal mass. This rare pathology is lacking level one evidence-based standardized treatment algorithms. The mainstay of treatment is surgical resection.

**Conclusion:**

For spermatic cord liposarcomas, the surgical approach is en bloc resection with radical orchiectomy aiming for R0 margins. Prognosis depends on tumor grade, anatomic site, and the ability to achieve a microscopically tumor negative resection. Despite our patient’s disease free status, prolonged surveillance with physical examination and cross sectional imaging is still warranted.

## Highlights

• Liposarcomas typically presents as bulky heterogeneous solid lesions that are larger/firmer than simple “cord lipoma.” Tumors range from 3 to 30 cm in diameter.
• Current controversy in management of liposarcomas pertains to the use of adjuvant chemotherapy or radiotherapy and whether these treatment options decrease recurrence/survival.
• We recommend an initial complete resection with wide margins without the use of adjuvant therapy with aggressive long-term surveillance.
• Based on extrapolated data from studies pertaining to sarcomas of the extremities, adjuvant radiation or chemotherapy may serve a role but further research is crucial.

## 1 Introduction

Malignant spermatic cord tumors comprise a rare group of pathologic entities with an annual incidence of only 0.3 cases per million [1]. The vast majority of tumors in this region are benign and comprise about 70–80% of masses identified. Among malignant tumors of the inguinal region, sarcomas are the most common type with liposarcomas accounting for 3–7% of spermatic cord tumors [2]. The major sarcomas identified are liposarcomas, leiomyosarcomas, malignant histiocytic fibroma, and fibrosarcomas, respectively from most to least common [3].

Regarding liposarcomas, five-year survival rates ranging from 15 to 85%, depending on tumor grade, anatomic site, and the ability to achieve a microscopically complete or R0 resection [4,5]. According to the World Health Organization, there are 5 histologic subtypes of liposarcomas: well differentiated, dedifferentiated, myxoid, round cell, and pleomorphic [2].

The inguinal canal in males is a small channel in the lower abdominal wall that contains the vas deferens, testicular artery and veins, lymphatic vessels, and nerves which compose the spermatic cord. This joins to the epididymis and testicle within the scrotum and links to the pelvic cavity. These structures can be involved in a wide array of pathologies, including neoplastic, congenital, and inflammatory etiologies which require a thorough history and physical exam to diagnosis [6]. The clinical manifestations of a malignant inguinal mass can be difficult to differentiate against benign lesions. A thorough history may help the clinician suspect malignancy with the history of growth, large size, and symptomatic presentation. Physical exam can usually differentiate between a simple hernia and hydrocele, but imaging is indicated when more sinister pathology is suspected [6].

The initial imaging modality of choice is ultrasound for its high sensitivity to characterize intra-testicular and extra-testicular lesions, ease of performance, and relatively inexpensive cost. Despite only suggesting the diagnosis in about 50% of cases, CT or MRI imaging remain critical tools to aid in diagnosis and surgical planning for suspected malignant lesions within the inguinal region [[2], [3], [4], [5], [6]].

Intraoperatively, liposarcoma typically presents as a bulky heterogeneous solid lesions that are larger and firmer compared to a simple “cord lipoma.” Tumors have been found to range from 3 to 30 cm in diameter [6]. On gross examination, the tumor can have a variable appearance with lobulated yellow-tan areas, indicative of the well-differentiated component and admixed with tan-gray-white fleshy or firm areas indicative of the dedifferentiated component [7]. Definitive diagnosis through histologic examination, immunohistochemistry, and cytogenetics remains the gold standard. Classic histology consists of a non-lipogenic, usually high-grade and cellular sarcomatous component adjacent to a well-differentiated liposarcoma. Murine double minute 2 (MDM2), a proto-oncogene, is amplified in dedifferentiated liposarcomas while negative in benign adipocytic tumors. Amplification is best demonstrated by strong, diffuse nuclear immunohistochemical staining [[4], [5], [6], [7], [8]]. In cases where tissue is limited, fluorescence in situ hybridization (FISH) can be used as an adjunct to detect MDM2 gene amplification with studies showing higher sensitivity and specificity over immunohistochemistry [9]. This case has been reported in line with the SCARE criteria [10].

## 2 Case presentation

**Fig. 1:**
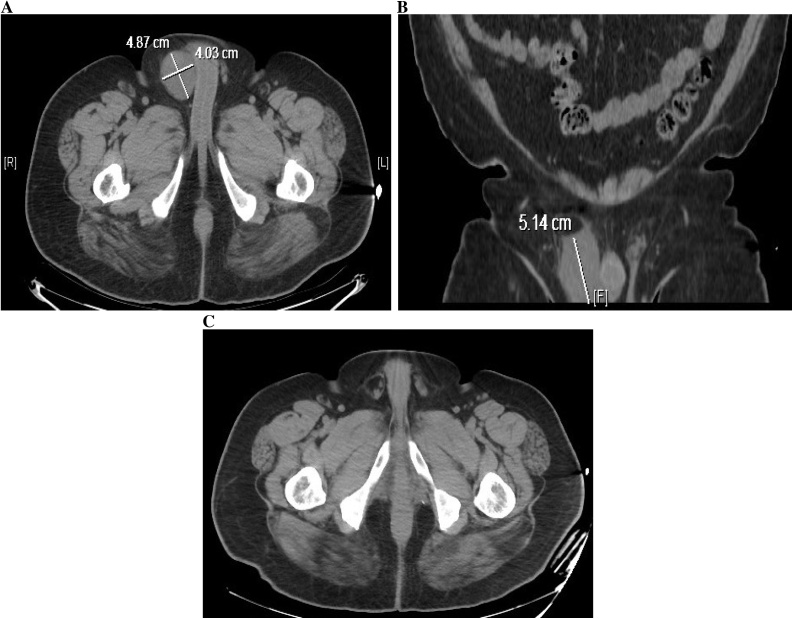
**A**: CT axial image illustrating a right inguinal canal soft tissue density measuring 4.87 × 4.03 cm. **B**: CT coronal image with right inguinal soft tissue density measuring 5.14 cm in length. **C**: CT axial image 22 months prior illustrating prominent fat in the right inguinal canal region surrounding the cord structures with no suspicious lesions.

A 59-year-old gentleman presented to outpatient clinic complaining of an increasing right groin mass with associated pain and discomfort. The patient denied weight loss or groin injury. No family history of cancer was noted. He was afebrile and vitals were within normal limits. On exam there was a grossly apparent large right inguinal mass measuring about 10 cm in diameter with no skin changes or signs of infection. A computerized tomography (CT) scan revealed a right inguinal canal soft tissue mass measuring 5.1 × 4.87 × 4.03 cm that was not present on previous imaging 22 months prior for an episode of nephrolithiasis (Fig. 1).

**Fig. 2:**
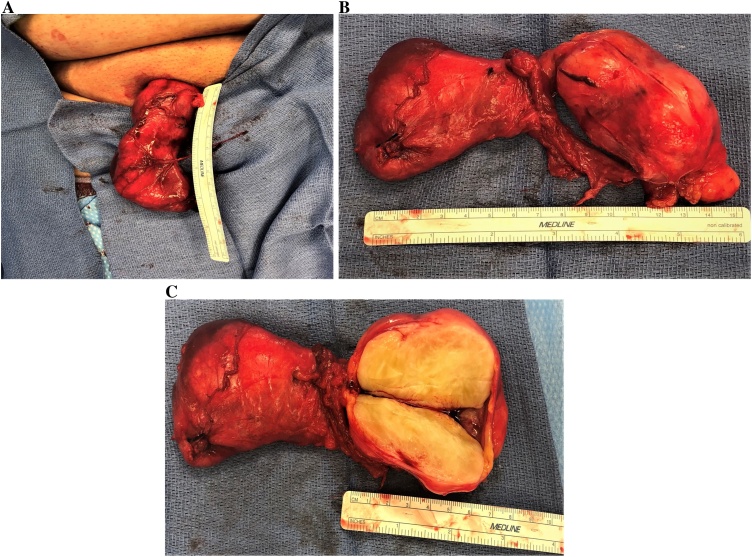
**A:** Right inguinal surgical approach with the spermatic cord lesion and right testicle in vivo. **B:** Gross image of the right inguinal mass excision with radical orchiectomy. The testicle is on the left and liposarcoma is on the right. **C:** Intraoperative image of the right inguinal mass excision with radical orchiectomy with the liposarcoma bisected illustrating the bulky heterogeneous solid lesion and typical yellow-tan appearance measuring 9 × 6 × 5 cm.

**Fig. 3:**
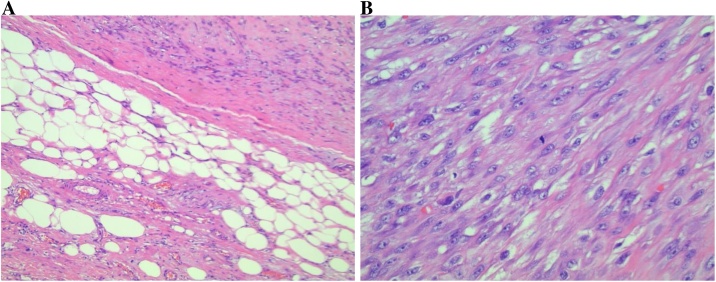
**A:** Hematoxylin and Eosin stained sections of the mass (200X) illustrating the well-differentiated component at the bottom, consisting of mature variably sized adipocytes with bands of fibrous stroma which contain occasional enlarged hyperchromatic nuclei. The dedifferentiated component is seen above, consisting of a cellular spindle cell proliferation. **B:** Hematoxylin and Eosin stained sections of the mass (400X) demonstrating the the nonlipogenic sarcoma aspect of the specimen, which is composed of closely packed high-grade plump fibroblast-like cells arranged in a fascicular pattern. Mitoses are easily identified.

Due to the size of the mass, location, associated pain, and previous CT scan there was concern for a spermatic cord malignancy and the patient was consented for a right inguinal exploration, resection, and possible radical orchiectomy. Intraoperative examination revealed a large soft tissue mass that was intimately attached to the spermatic cord. There was high concern for malignancy prompting an en bloc resection of the mass with a radical orchiectomy to ensure negative margins (Fig. 2). Histopathological tissue analysis revealed a grade 2 dedifferentiated liposarcoma measuring 9 × 6 × 5 cm and weighed 100 g (Fig. 3). Margins were microscopically negative at 5 cm. Fluorescence In Situ Hybridization (FISH) showed positive immunoreactivity amplification of proto-oncogene protein murine double minute 2 (MDM2) confirming the diagnosis. The patient recovered well and was discharged home on postoperative day one. On 6 month follow-up, the patient continues to do well with no signs of recurrence or metastasis on surveillance imaging.

## 3 Discussion

Despite literature accounts of surgeons’ experience with inguinal malignant neoplasms such as liposarcomas, this pathology is lacking level one evidence-based standardized surgical management algorithms. Most of the data published are case reports, surgeon experience, and retrospective analysis of hospital specific outcomes. This pathologic entity happens too infrequently for any single institution to accumulate enough cases to prospectively undertake randomized control trials. In one of the largest cohorts of spermatic cord tumors, investigators at Harvard Medical School analyzed 362 patients and concluded an annual incidence of 0.3 cases per million which did not change over time. Rodriguez et al. observed worse outcomes with undifferentiated tumor grade, distant disease, positive lymph nodes, and leiomyosarcoma or histiocytoma cell histology [1].

Liposarcomas, as with most sarcomas invade through local extension of the mass and rarely spread via lymphatic routes. High grade subtypes are associated with higher rates of recurrence and hematogenous spread to the bone and lungs [2]. It is therefore imperative and widely agreed upon that the treatment based on the available limited literature proceeds with en bloc resection of the mass along with a radical orchiectomy and high ligation of the spermatic cord with the main goal of achieving negative margins or R0 resection [2].

The current literature regarding management is limited with only case reports and single-institution series. When a spermatic cord tumor is suspected the only generally accepted treatment entails wide resection with a radical orchiectomy and high ligation of the spermatic cord for complete tissue analysis. The utility of preoperative fine needle aspiration (FNA) cytology has not been clearly established [11]. Complete tissue analysis with a wide resection and radical orchiectomy is crucial due to the aggressive nature of these tumors with a 5 year mortality rate as high as 85% [4,5]. The few cases with a preoperative biopsy proven malignancy come from unique situations with an initially missed or delayed diagnosis. Chalouhy et al. reports a case of an inguinal liposarcoma that was originally diagnosed and scheduled for as an indirect hernia repair. Intraoperatively the mass was identified and an incisional biopsy was taken. The resection was delayed in order obtain consent for the resection and radical orchiectomy [2]. More research into the utility of preoperative histopathological specimen examination for spermatic cord tumors are needed along with obtaining the sensitivity and specificity for this diagnostic tool.

Due to the rarity of spermatic cord tumors and the overwhelming majority being sarcomas, much of recommendations are extrapolated from the general surgical principles of sarcoma therapy and testicular tumors. According to the American Urology Association when a clinician encounters a testicular lesion suspicious for malignant neoplasm a radical inguinal orchiectomy is recommended. Testis-sparing surgery is not recommended and transscrotal biopsy or orchiectomy is discouraged. (Strong Recommendation; Evidence Level: Grade B) [12].

Regarding the sarcoma literature, tumors of all grades tend to infiltrate local tissues which increases the difficulty of completing a complete resection. An inadequate resection promotes the seeding of tumor through the operative site with recurrence rates as high as 50% in cases that employed a less aggressive, simple excision. Therefore, an aggressive surgical approach has been recommended in the management of spermatic cord tumors [11].

The current controversy in management is in regards to the use of adjuvant chemotherapy or radiotherapy and whether these additional treatment options actually decrease recurrence and ultimately improve survival. Unfortunately, due to the rarity of this disease there is limited data into this matter with no randomized control studies available in the literature. Coleman et al. from Memorial Sloan-Kettering Cancer Center describe their 20 year surgical experience with spermatic cord sarcomas in 47 patients. In their patient cohort, 21 (45%) were treated with adjuvant radiation and 9 (19%) received chemotherapy. However, investigators were unable to demonstrate a therapeutic effect with these therapies. Interestingly, in 21 patients who underwent reoperative wide resection after a prior incomplete resection, authors concluded a trend toward improved disease-free survival (p = 0.059). Additionally, disease-free survival over time was shorter when there were positive surgical margins at first and second resection (p < 0.05), illustrating the importance of an aggressive surgical approach to achieve R0 resection. Coleman suggests that despite adjuvant radiotherapy not shown to increase long-term survival in extremity lesions but shown to decrease local recurrence perhaps there is a benefit in select cases of spermatic cord sarcomas in high-risk patients with multiple recurrences, positive margins, and high grade tumors [13].

Based on studies of sarcomas of the extremities, adjuvant radiotherapy may play a role in recurrent or residual liposarcomas of the spermatic cord. In France, Khanfir et al. showed a significant decrease in local recurrence in patients with margins less than 10 mm (p = 0.005) and in patients with residual tumor cells after re-excision (p = 0.001). However, investigators were unable to show significant influence on 5- and 10-year overall survival [14].

In Italy, Frustaci et al. was able to show a survival benefit for intensive chemotherapy for high grade (grade 3 or 4) sarcomas of the extremities greater or equal to 5 cm or any size recurrent tumor. Authors found a significant benefit in median overall survival of 75 months for treated and 46 months for untreated patients (p = 0.03). In addition, an absolute benefit from chemotherapy was 13% at 2 years and increased to 19% at 4 years (p = 0.04) [15].

In an extensive literature review, Morozumi et al. found that only 326 cases of spermatic cord liposarcomas have been reported with only 15% of liposarcomas being of the dedifferentiated subtype. Authors describe a case of dedifferentiatted liposarcomas that was diagnosed at the 7th resection of recurrence, indicating the difficulty and importance of making the initial diagnosis with subsequent wide and complete resection with negative microscopic margins [16]. Due to the multiple recurrences and grade of the tumor, authors proceeded to treat with adjuvant chemotherapy and concluded stable disease at 8 months follow up. Investigators suggest considering adjuvant chemotherapy and/or radiotherapy in cases of high risk for recurrence.

In only the 14th reported case in Japan, Tobiume et al. recently published a similar case of a dedifferentiated liposarcoma of the spermatic cord with a positive surgical margin. An additional wide resection to achieve an R0 resection without adjuvant therapy was successfully carried out with patient doing well 12 months postoperatively without signs of recurrence [8].

In our case, due to the complete resection of the mass with negative 5 cm margins and this being the initial occurrence with no clear guidelines or randomized control trials recommending adjuvant therapy it was decided to conclude treatment and proceed with aggressive clinical follow-up. Authors from Johns Hopkins Hospital recently published a similar case of a well differentiated liposarcoma of the spermatic cord and showed a successful outcome through employing the same operative strategy. Despite being a relatively less aggressive tumor, uncertainty still remained in regards to optimal treatment. Chalouy et al. notes the lack of available literature with no gold standard of treatment and how the current management of this rare pathology is based on case reports of surgeons’ experiences [2].

The accepted treatment for liposarcomas of the spermatic cord encompasses an en bloc resection of the mass along with a radical orchiectomy and high ligation of the spermatic cord with the main goal of achieving a R0 resection. Based on extrapolated data of the available studies pertaining to sarcomas of the extremities, adjuvant radiation or chemotherapy may serve a vital role in high risk situations. Further research into this matter is crucial. Despite our patient’s disease free status, prolonged surveillance with physical examination and cross sectional imaging is still warranted.

## 4 Conclusion

We present a rare case of a dedifferentiated liposarcoma of the spermatic cord that was successfully treated with an R0 resection. This case highlights the importance of maintaining a high index of suspicion coupled with a thorough history and physical examination when encountering an enlarging inguinal mass. Despite accounts of surgeons’ experience with liposarcomas of the spermatic cord, this rare pathologic entity is lacking level one evidence-based standardized treatment algorithms. Initial treatment encompasses an en bloc resection of the mass along with a radical orchiectomy and high ligation of the spermatic cord with the main goal of achieving a negative margin or R0 resection. More research is needed in the matter of adjuvant radiation and chemotherapy to provide surgeons with evidence-based standardized approach to ensure optimal patient outcomes.

## Declaration of Competing Interest

None.

## Funding

None.

## Ethical approval

This report was conducted in compliance with ethical standards. Informed written consent has been obtained and all identifying information is omitted.

## Consent

Informed written consent has been obtained and all identifying information is omitted.

## Author contribution

YS, AE, DB, MM, DK, AA, JW – Conception of study, acquisition of data, analysis and interpretation of data, drafting the article, and revision of article. JW – Management of case.

YS, AE, DB, MM, DK, AA, JW – Approval of the final version for submission.

## Registration of research studies

This is a case report study.

## Guarantor

Jason Wolf.

## Provenance and peer review

Not commissioned, externally peer-reviewed.
